# Bioecological correlates of learning outcomes: testing the moderating role of obesity in academic performance

**DOI:** 10.3389/fpsyg.2026.1916078

**Published:** 2026-09-23

**Authors:** Izzettin Aydogan

**Affiliations:** Faculty of Education, Van Yüzüncü Yıl University, Van, Türkiye

**Keywords:** academic penalty, body image, compensatory effort, inclusive learning environments, weight stigma

## Abstract

**Introduction:**

This study examined the moderating role of obesity in adolescent academic performance using the Bioecological Model of Human Development as the theoretical framework.

**Methods:**

Data from 2,127 adolescent students in the PISA 2022 dataset were analyzed using multi-group path analysis.

**Results:**

Sixteen of the seventeen associations examined were of comparable magnitude in the two groups, and the joint test of all seventeen provided no evidence of moderation. Contrary to expectations, body image was negatively associated with achievement in both groups, meaning that adolescents more satisfied with their bodies performed less well. The association was more than twice as strong among obese adolescents, the only between-group difference observed, and it did not persist after adjustment for multiple comparisons. Psychosomatic symptoms also showed a positive relationship with achievement, contrary to what the literature reports, reaching significance among obese adolescents alone, while perceived school safety reached significance only among their non-obese peers; in neither case did the groups differ. Curiosity, emotional control, and perseverance were positively related to achievement in both groups, and social connections, cooperation, and school risk were negatively related.

**Discussion:**

These findings locate the difference associated with obesity in adolescents' body-related self-perception rather than in the relational or institutional resources available to them. Educational strategies that address weight-inclusive environments and bias training may therefore complement traditional health-focused interventions in supporting these students.

## 1 Introduction

In adolescence, obesity has evolved beyond being merely a clinical factor that threatens physical and metabolic health; it has become a multidimensional global problem that affects individuals' psychosocial wellbeing, cognitive functions, and educational outcomes (Tekchandani et al., 2026). In 2022, 8% of children and adolescents aged 5 to 19 were living with obesity, compared with 2% in 1990, which corresponds to more than 160 million young people worldwide (NCD Risk Factor Collaboration (NCD-RisC), 2024). An expanding body of research reveals that adolescents with obesity and overweight exhibit lower academic performance, higher rates of school absenteeism, and a marked decline in overall school motivation compared to their normal-weight peers (Ma et al., 2020; Včelarová et al., 2022). However, this disadvantage, broadly conceptualized as an academic penalty in educational sociology and psychology, cannot be explained solely by a lack of cognitive capacity (Branigan, 2017). Rather, this process is the result of debilitating dynamics, including body image dissatisfaction, weight-based stigma, peer rejection, and social exclusion, which can undermine adolescents' learning. Behind this academic penalty lie the psychological and social barriers that adolescents with obesity must face every day in the school environment. Exposed to peer bullying, ridicule, and often implicit prejudice, these students gradually withdraw from classroom interactions, and their sense of belonging to the school weakens (Langford et al., 2022; Puhl et al., 2020). A lack of social acceptance and chronic anxiety about being judged cause the individual to divert their cognitive resources (e.g., working memory and attention) toward managing these social threats rather than academic tasks (Tekchandani et al., 2026). In addition, body image dissatisfaction and declining self-esteem weaken adolescents' resilience to stress, triggering psychosomatic symptoms and depressive tendencies that accelerate academic burnout (Emon et al., 2024; Sikdar, 2021). In other words, obesity is not merely a physical characteristic of the student; it can also shape the quality of the student's relationships with their environment, teachers, and learning materials.

Despite the complexity of this issue, conceptual and methodological gaps are evident in the current literature focusing on the educational consequences of obesity. A large portion of recent studies has been limited to examining direct correlations between static body mass indices (BMI) and grade point averages, or reducing the issue solely to the physical activity/cognitive development axis (Guimarães et al., 2023; Hermassi et al., 2024). However, an adolescent's academic performance is shaped by the simultaneous interaction of internal resilience resources—such as curiosity, perseverance, and empathy—perceived social support from parents and teachers, and the sense of safety provided by the school environment (Oraibi et al., 2024). For adolescents living under the psychosocial pressure associated with obesity, these social, emotional, and institutional resources may carry different weight than they do for their normal-weight peers. Crosnoe and Muller (2004) found the negative association between obesity risk and achievement to be stronger in school contexts where obesity was more likely to be stigmatized, and Roorda et al. (2011) report that supportive relationships matter more for students who are academically at risk. Yet, the literature lacks comprehensive empirical investigations that examine the moderating role of obesity in these psychosocial and environmental support processes using large-scale, comparative datasets. This research aims to overcome the narrowness of the literature by examining the statistical differences in the associations of personal, emotional, and institutional characteristics with the academic performance of adolescents with obesity and their non-obese peers. The main problem of the research is whether obesity differentiates the socio-emotional skills, relational networks, and institutional climate surrounding the learning process, and in what direction these multidimensional interactions are reflected in academic performance. This study, designed using the PISA 2022 dataset examines how the associations of socio-emotional skills such as perseverance, curiosity, and stress resistance, relational networks shaped around family and teacher support, and institutional climate characteristics such as safety and school belonging with academic performance differ for adolescents with obesity compared to their normal-weight peers (see Figure 1). The results are intended to clarify where obesity does and does not make a difference in education, and to provide a theoretical and empirical basis for the evidence-based design of education policies, inclusive school climate strategies, and psychosocial support mechanisms, going beyond purely medical interventions.

**Figure 1:**
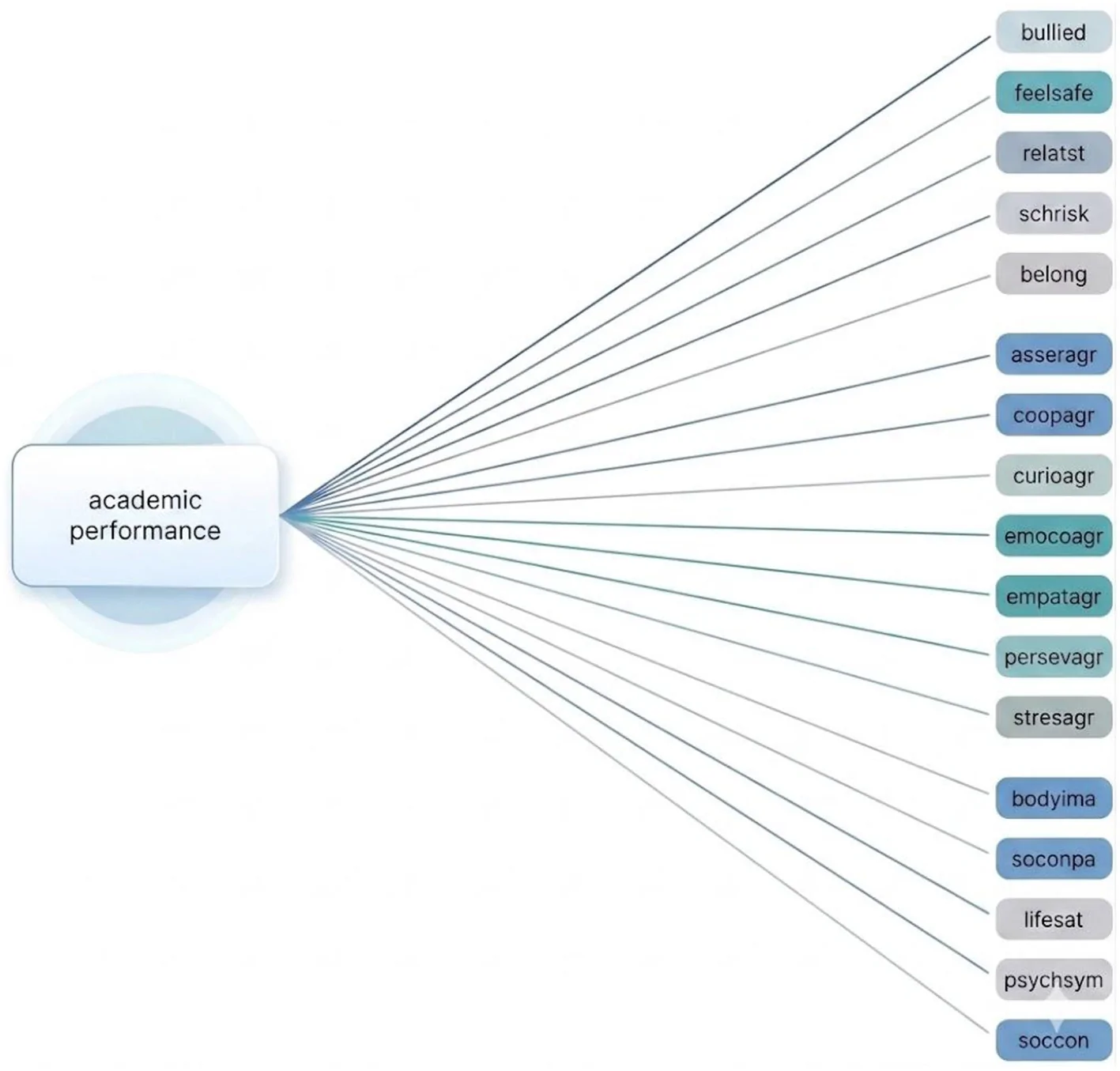
Theoretical model.

## 2 Theoretical background

This section outlines the theoretical basis of the study in two steps. First, Bronfenbrenner and Morris's bioecological model is presented as the overarching framework that locates obesity within the nested systems shaping adolescent development. Second, because the bioecological model provides a general account of how proximal processes shape development, it is complemented by three perspectives that speak more specifically to the case of obesity: Stereotype threat, identity threat and coping, and thwarted belongingness. These perspectives inform the interpretation of the findings, though they are not directly examined in the analyses.

### 2.1 Bioecological model of human development

Bronfenbrenner's ecological model, developed in 1979, describes human development through the nested environmental systems in which the individual is situated. It has been criticized, however, for not explaining the developmental process itself: how development occurs, what shapes it, and under what conditions the individual interacts with the environment (Darling, 2007). Accordingly, the model was restructured around four core components, namely Process, Person, Context, and Time, collectively known as the PPCT Model (Bronfenbrenner and Morris, 2006). The PPCT Model treats development as the product of continuous, reciprocal interactions (Process) between an individual's biological and psychological characteristics (Person) and their environment (Context), unfolding within a historical and developmental timeframe (Time).

A central contribution of the model in terms of research design is its shift in analytical focus from a static description of environmental structures to the proximal processes between the individual and their environment. Proximal processes encompass the regular, reciprocal, and increasingly complex direct interactions that a developing individual establishes with their immediate environment, such as parents, peers, teachers, objects, and symbols (Bronfenbrenner and Morris, 2006). Research has consistently shown that developmental outcomes such as academic performance are shaped by the quality and regularity of these proximal processes (Gutman and Schoon, 2013). The *Person* component is the most relevant part of the model for this study. Bronfenbrenner and Morris (2006) defined the biological, physiological, and morphological characteristics of the developing individual, including body weight and composition, as developmental instigators that shape the direction, intensity, and nature of interactions with the environment. Within this framework, obesity is more than a clinical health condition: it can alter how proximal processes operate across the layers of the ecological system. It is therefore treated as a moderator variable in this study. The *Time* component, in turn, incorporates the developmental context of adolescence into the framework. Adolescence is a critical stage marked by intense biological changes and a profound restructuring of interactions with the social environment (Benner et al., 2017). During this period, adolescents deal at once with shifting family dynamics, a changing peer culture, and an increasingly institutionalized school climate. Therefore, complex constructs of this kind are best explained by the dynamic interactions between individuals' proximal and distal environments (Gutman and Schoon, 2013).

### 2.2 Positioning the research variables within the ecological system layers

The 17 predictor variables obtained from the PISA 2022 background questionnaires were classified under three ecological layers in line with the *Context* component of the PPCT model: microsystem, mesosystem, and exosystem. This classification both clarifies the theoretical positioning of the variables within the research model and provides the necessary framework for systematically testing the moderating role of obesity across these layers.

#### 2.2.1 Microsystem: self-perception, internal processes, and psychological dynamics

The microsystem is the innermost circle of development within which the developing individual engages in face-to-face interactions and directly makes sense of their experiences (Bronfenbrenner and Morris, 2006). During adolescence, the microsystem encompasses not only the physical environment but also the individual's inner world, psychological characteristics, emotional regulation capacity, and the personal skills they draw on when relating to others. The general social and emotional characteristics and the intrapersonal wellbeing variables examined in this study, derived from the PISA 2022 background questionnaires, are categorized under this layer.

The first of these two groups comprises the general social and emotional characteristics. Academic perseverance (Persevagr) reflects determination in achieving academic goals and curiosity (Curioagr) indicates a desire for intellectual discovery, while cooperation (Coopagr), empathy (Empatagr), and assertiveness (Asseragr) describe, respectively, a tendency toward collaboration, the capacity to understand others' emotions, and proactivity in social settings. Two further indices belong to the emotional regulation cluster: stress resistance (Stresagr), the ability to remain calm under pressure and recover from adverse situations, and emotional control (Emocoagr), the ability to regulate sudden emotional reactions. These seven characteristics have been associated with academic outcomes in adolescence (Durlak et al., 2011; Soto et al., 2024). The second group comprises the intrapersonal wellbeing variables, which concern how adolescents perceive and evaluate their own physical and psychological state. Body image perception (Bodyima) indicates how positively one perceives their body, life satisfaction (Lifesat) represents a holistic assessment of life, and psychosomatic symptoms (Psychsym) cover the physical complaints linked to psychological distress (Myers and Rosen, 1999; Puhl and Heuer, 2009).

#### 2.2.2 Mesosystem: immediate social environment, relational networks, and inter-system interactions

The mesosystem describes the dynamic interaction between multiple microsystems, including the family and the school environment, in which the individual actively participates (Bronfenbrenner and Morris, 2006). The consistency of social support networks across different life contexts may buffer risks between systems; strong ties in one context have the capacity to offset fragility in another. The longitudinal study by Wang and Eccles (2012) provides a critical reference point for this level: when examining the effects of parental, teacher, and peer support on school engagement, it becomes evident that these sources are not equivalent and evolve over the course of developmental stages. While teacher and parent support act as the primary predictors of emotional engagement such as school identity, peer bonds become the determining factor for behavioral outcomes; this finding suggests that mesosystemic dynamics have a multidimensional and time-dependent structure rather than a static one. Accordingly, the support variables at this layer are examined together, since parental, peer, and teacher support work across the home and school contexts to shape how the adolescent functions.

The first of these three sources is the family. The quality of the parent-child relationship and emotional closeness (Soconpa) form the foundation of out-of-school support systems. According to Wang and Sheikh-Khalil (2014), this support encompasses not only emotional bonds but also academic socialization, in which parents discuss educational expectations, career plans, and schoolwork with their children, and such interactions have been associated with higher behavioral and emotional engagement at school. Social connections (Soccon) broadens this field beyond the household, measuring the ease with which adolescents discuss their anxieties and concerns with parents, siblings, and peers (Smetana et al., 2006). The academic and emotional support perceived from teachers (Relatst) represents the third source, a human capital interaction located within the school itself. The meta-analysis by Roorda et al. (2011) indicates that positive teacher-student relationships serve as a more vital buffer for academically at-risk and disadvantaged students. Since obese adolescents, who bear the burden of stereotypes, fall precisely into this at-risk group, teacher support may be particularly relevant for them.

#### 2.2.3 Exosystem: organizational environment and school climate

The exosystem refers to the institutional and environmental context in which adolescents cannot directly participate in processes but are influenced by the decisions made within that context and its overall atmosphere (Bronfenbrenner and Morris, 2006). The security structure of the school grounds and the nature of peer culture are structural factors that take shape beyond the adolescent's control but can affect their motivation to learn and their academic focus. These indicators are therefore treated as perceived features of the institutional climate, that is, as conditions the adolescent is exposed to rather than ones they help create.

Four indices are located at this layer. Two of them describe conditions that support the institutional climate. The sense of belonging to school (Belong) represents a balance between feelings of social acceptance and exclusion, and perceived school safety (Feelsafe) reflects the subjective perception of safety in various areas of the school; both have been associated with learning outcomes. According to the belongingness hypothesis of Baumeister and Leary (1995), when the need to belong is left unmet, individuals devote a significant portion of their cognitive and emotional capacity to managing the threat of social exclusion. The remaining two indices describe conditions that work against it. Exposure to peer bullying (Bullied) and school safety risks (Schrisk) cover incidents such as fights and threats on school grounds, which have been associated with disruption of the learning process (Cornell et al., 2016).

### 2.3 Moderating role of obesity: three complementary theoretical perspectives

Within the PPCT model, obesity acts as a Person characteristic that can reshape how proximal processes operate, though the model itself does not explain why this restructuring affects some adolescents more than others. To address this, three complementary theoretical perspectives are drawn upon: stereotype threat, identity threat and coping, and weight-based social exclusion and thwarted belongingness. These perspectives are treated as interpretive frameworks rather than as a causal sequence: each offers a plausible account of how weight-based stigma might relate to learning, and together they provide a richer reading of the moderation examined in this study.

#### 2.3.1 Stereotype threat

The first perspective concerns how obesity may create a cognitive load in an academic setting. Steele and Aronson (1995) defined stereotype threat as a situation in which an individual faces the risk of personally confirming a negative stereotype attributed to their own group. The basic process is as follows: when an individual perceives this threat, they divert a portion of their cognitive capacity away from the task at hand and toward managing the threat; working memory, cognitive flexibility, and attention are divided, leaving the remaining capacity insufficient for the academic task. It has been extensively confirmed that this process extends far beyond race-based stereotypes and applies to many stigmatized identities, including weight-based stereotypes (Puhl et al., 2020).

Within the PPCT model, this division of cognitive capacity helps explain why a negative school climate may affect obese adolescents more than their normal-weight peers. They are already spending mental energy on managing stereotype threat, so the same environment leaves them with less to give to schoolwork. A key point in this account is that the cognitive dissonance associated with stereotype threat may gradually affect the adolescent's self-image. An obese adolescent who repeatedly encounters this threat in an academic setting may begin to internalize failure through a self-assessment that aligns with the stereotype, which connects to the second perspective, identity threat.

#### 2.3.2 Identity threat and coping

The second perspective addresses how the accumulation of *stereotype threat* triggers a restructuring at the identity level. The identity threat model of stigma by Major and O'Brien (2005) posits that a stigmatized individual can develop three primary strategies to cope with this threat: (a) academic disengagement—separating academic outcomes from one's self-concept, thereby limiting the damage that failure inflicts on self-worth; (b) compensation—attempting to overcome the stigma by increasing the drive for success and clinging tightly to social support resources; (c) assimilation—reducing visibility by conforming to the social group. Which path is chosen is not random; it is a direct function of the individual's ecological resource pool, particularly the quality of their social support networks (Major and O'Brien, 2005). At this point, Crocker and Major (1989) emphasize that stigmatized groups can employ the strategy of selective valuing/devaluing as a coping mechanism against stigma and as a form of psychological armor. Where obese adolescents downplay school and learning, this may reflect not simply a loss of motivation but a self-protective strategy based on a decision to diminish the personal importance of the academic domain from which they are excluded, in order to preserve their self-esteem. Which strategy an adolescent chooses depends directly on the quality of resources available within the microsystem and mesosystem.

Within the PPCT model, the relational bond established with parents has been theorized as a mesosystemic resource that may support a compensatory response to identity threat. Where such support is available, stereotype-related pressure could, in principle, mobilize rather than dampen academic motivation, as the effort to overcome stigma comes into play (Crocker and Major, 1989). Body image is understood here as a distinct element of this process. Rather than a purely aesthetic concern, a negative body image may reflect the internalization of weight-based stigma — the gradual shaping of self-perception by societal stereotypes — and, on this reading, could represent one of the more consequential psychosocial correlates of academic outcomes among obese adolescents. Whether this pressure leads to disengagement or to compensation is not something the theory settles in advance. Because the choice of coping strategy depends on the adolescent's social bonds, this leads to the third perspective: weight-based social exclusion and thwarted belongingness.

#### 2.3.3 Weight-based social exclusion and thwarted belongingness

The third perspective considers how the previous two processes take shape within a social context. The belongingness hypothesis of Baumeister and Leary (1995) posits that interactions within the framework of frequent, non-negative, and ongoing relational bonds constitute a fundamental human motivation; it further suggests that when this need is not met, cognitive processes, emotional regulation, and motivational resources are severely disrupted. Obese adolescents face this vulnerability to a disproportionate degree: A large-scale longitudinal study by Lumeng et al. (2010) revealed that obese children face a significantly higher risk of peer bullying, regardless of gender, race, socioeconomic status, social skills, or academic performance. Puhl and Heuer (2009), meanwhile, note that exclusion in educational settings is not limited to peers; it also manifests as lower expectations from teachers and educators toward obese students, thereby exposing the student to a dual risk of exclusion (from both peers and teachers).

Experiences of exclusion and insecurity in the school environment create a feedback loop that deepens the psychological burden generated by the previous two processes. For a normal-weight adolescent, a negative school climate is a contextual adversity; for an obese adolescent, however, the same experience is an intersection where three processes are simultaneously activated: the concretization of *stereotype threat*, the reinforcement of *identity threat*, and the fragilization of the need for belonging (thwarted belongingness). Moreover, weight-based exclusion may affect academic functioning through psychological as well as physiological channels. Research by Schvey et al. (2014) shows that cortisol reactivity is elevated in individuals exposed to weight stigma, regardless of their weight status, and such HPA-mediated stress responses have been associated with reduced learning, memory, and executive functioning (Puhl et al., 2020). Together, these findings suggest that these academic difficulties reflect more than low motivation.

Considered together, these perspectives suggest a pattern in which the narrowing of coping strategies under stereotype threat may increase the need for social bonds, while exclusion and insecurity in the school environment can make those bonds feel fragile. This pattern is proposed as an interpretive account of the observed group differences rather than as a causal process tested in the present study.

## 3 Methods

### 3.1 Data and participants

This study was conducted using data from the PISA 2022 assessment, which is publicly available on the official website of the Organization for Economic Co-operation and Development (OECD; https://www.oecd.org/en/data/datasets/pisa-2022-database.html#data). The data reflect students' responses to the items included in the Student Questionnaire and Well-Being Questionnaires.

Although the PISA assessment encompasses data from numerous participating economies, the administration of the Well-Being Questionnaire—which contains the core microsystemic variables of this study, such as body image, stress resistance, and psychosomatic symptoms—along with the collection of objective Body Mass Index (BMI) data, was restricted to a limited number of countries. Consequently, the study sample was specifically drawn from three countries (Hong Kong, Ireland, and New Zealand) that provided comprehensive and complete datasets across all the psychosocial scales and anthropometric measurements required to test the proposed bioecological model. In all, the research group consists of 2,127 adolescents (1,035 female, 1,092 male) aged 15. Since the study focused on the association between obesity and learning outcomes, the primary criterion in designing the study group was to identify individuals who were either non-obese or at the obese weight level. In this context, BMI values calculated from data provided in the PISA dataset were used as a reference for selecting participants. Participants were identified based on BMI values classified as obese according to the gender and age criteria established by the World Health Organization (n.d.). BMI values were calculated for 91,074 students, and 10,448 records were complete on all study variables; 709 of these students met the criterion for obesity. Since gender and socioeconomic status (ESCS) are two key demographic characteristics associated with obesity and academic outcomes (Branigan, 2017; Morales-Suárez-Varela et al., 2018; Nam and Park, 2021), and since the study focused on examining the moderating role of obesity, gender and ESCS were treated as covariates. Accordingly, non-obese controls were selected through propensity-score matching at a 1:2 ratio. The propensity score was estimated by logistic regression of obesity status on ESCS. Matching proceeded by nearest-neighbor selection without replacement, with country and gender imposed as exact-matching constraints, and yielded 709 matched sets comprising 709 obese and 1,418 non-obese adolescents. Estimated scores ranged from 0.047 to 0.112 (*M* = 0.070), a span narrow enough that no caliper was required to prevent poorly matched pairs. A 1:2 ratio was chosen because the obese group was small relative to the non-obese pool. Matching and weighting address different concerns: matching removes the demographic differences between the two groups, whereas the sampling weights and the BRR variance estimator determine how far the estimates extend beyond the matched sample. The ratio was therefore set at a level that achieved covariate balance rather than one that maximized sample size. Balance was assessed through standardized mean differences before and after matching. Prior to matching, obese adolescents came from less advantaged backgrounds than the non-obese pool (standardized difference = −0.197) and were unevenly distributed across the three systems, with Hong Kong accounting for 43.3% of the non-obese pool but only 22.7% of the obese group. After matching, the ESCS difference fell to −0.004 with a variance ratio of 1.01, and the distributions of country and gender were identical in the two groups, with all standardized differences well within the conventional ±0.10 threshold (Hennessy et al., 1999; Stuart, 2010).

The reduction from 91,074 students with calculated BMI values to 10,448 complete records reflects the structure of the PISA 2022 questionnaire design rather than individual non-response. Of the twelve economies in which BMI data were collected, nine did not administer at least one of the questionnaire modules required by the model. Cooperation, perseverance, assertiveness, and stress resistance were unavailable in the United Arab Emirates, Panama, and Saudi Arabia; cooperation was also unavailable in Brazil and Slovenia; empathy was unavailable in Brazil, Mexico, Panama, and Slovenia; and school risks and perceived school safety were unavailable in Spain, Hungary, and Macao. Coverage was complete for all seventeen indices only in Hong Kong, Ireland, and New Zealand, which is why the analysis is confined to these three systems. Within them, item-level missingness ranged from 1% to 8% across the seventeen indices, and 10,448 of the 12,156 eligible students, or 86%, had complete records on all study variables. Complete-case analysis was therefore retained, since the dominant source of missingness operates at the level of the questionnaire design. Within the three systems, however, the students retained were somewhat higher-achieving than those excluded, by roughly half a standard deviation (*M* = 529.5 vs. 483.9), and came from slightly more advantaged backgrounds. This bears on how far the estimates extend to the full student population of the three systems rather than on the comparisons between the two weight groups, since both groups are drawn from the same retained pool.

### 3.2 Variables

The seventeen predictor variables were selected as three coherent blocks of psychosocial indices that PISA 2022 provides, each corresponding to one layer of the bioecological model. The first block comprises the general social and emotional characteristics, which PISA measures through indices built on the OECD's Big Five–based framework of social and emotional skills (perseverance, curiosity, cooperation, empathy, assertiveness, stress resistance, and emotional control). These skills were prioritized because large-scale evidence, including the OECD's own PISA 2022 analyses, identifies task-related and openness skills such as persistence and curiosity as among the social-emotional characteristics most strongly associated with achievement (OECD, 2024; Soto et al., 2024). The second block covers wellbeing indices, namely body image, life satisfaction, psychosomatic symptoms, and the quality of social connections with parents and peers, which capture the affective and relational dimensions of adolescent functioning that recent work links to academic outcomes (Wang and Sheikh-Khalil, 2014). The third block consists of school culture and climate indices, including student–teacher relationships, sense of belonging, bullying, feeling safe, and school risk, which are dimensions repeatedly shown to shape achievement and engagement (Roorda et al., 2011). Together, these blocks operationalize the individual, relational, and institutional layers of the bioecological model within the constraints of the data available in the PISA 2022 Student and Well-Being Questionnaire.

As summarized in Table 1, these indices were reorganized under the micro-, meso-, and exosystem layers of Bronfenbrenner's model in line with the study's theoretical framework. The predictor indices were derived from scale batteries consisting mostly of 4-point Likert-type scales (or, in the case of life satisfaction, an extended 0-to-10 format) and typically comprising 3 to 9 items, converted into continuous WLE (Warm's Weighted Likelihood Estimates) scores in accordance with item response theory (IRT) principles. The dependent variable, academic performance, was derived from the ten plausible values that PISA provides for each of the three cognitive domains (reading, mathematics, and science). Rather than reducing these to a single score, the plausible values were carried through the analysis and combined using Rubin (1987) rules, so that the measurement uncertainty inherent in the plausible-value methodology was reflected in the estimated standard errors (OECD, 2024; Rutkowski et al., 2010). For descriptive presentation, the resulting performance estimate was standardized to a mean of 0 and a standard deviation of 1. Body Mass Index (BMI) values, used as a physical indicator, served to define the study's moderator variable (obese and non-obese).

**Table 1:** Variables.

Variables	Construct	Features of variables
Outcome variable		
ZPV	Academic Performance	Overall achievement level of students in reading, mathematics, and science, derived from ten plausible values
Predictor variables		
School culture and climate		
Relatst	Student-teacher relationships	Quality of perceived academic and emotional support from teachers
Belong	Sense of belonging	Student's level of social acceptance and integration at school
Bullied	Being bullied	Frequency of physical, verbal, or social harassment experienced at school
Feelsafe	Feeling safe at school	Students' subjective perception of safety within the school environment
Schrisk	School risks	Intensity of tangible risks on school property, such as vandalism, fights, and threats
General social and emotional characteristics		
Persevagr	Perseverance	Tendency to finish what is started, exert extra effort during challenges/boredom, and persist despite obstacles
Curioagr	Curiosity	Desire to be curious about different things, ask questions, develop hypotheses, and learn how things work
Coopagr	Cooperation	Level of liking to help others, working better as part of a team, and enjoying cooperation with classmates
Empatagr	Empathy	Ability to sense others‘ feelings, understand different perspectives and anticipate others' needs
Asseragr	Assertiveness	Level of being comfortable taking the lead, convincing others, and speaking up about things that matter
Stresagr	Stress Resistance	Capacity to not get nervous/panic easily, work under pressure, and recover quickly from bad events
Emocoagr	Emotional Control	Ability to keep emotions under control, not get mad/upset easily, and avoid unpredictable mood swings
Wellbeing		
Bodyima	Body image	Adolescent's level of satisfaction with their overall look, weight, body, and how clothes fit
Soconpa	Social connection to parents	Frequency of parents providing needed help, trying to understand worries, and making the teen feel better when upset
Lifesat	Life satisfaction	Cognitive evaluation made by the adolescent regarding their overall life satisfaction on a 0–10 scale
Psychsym	Psychosomatic symptoms	Frequency of physical complaints in the past 6 months, such as headaches/stomach/back pains, feeling depressed, sleeplessness, or dizziness
Soccon	Social connections	Level of ease with which the adolescent can talk about things that bother them with their father, mother, siblings, or peers

### 3.3 Data analysis

The study was designed in two stages to comparatively examine the associations of seventeen predictors with academic performance in groups of obese and non-obese adolescents and to determine the extent to which these associations differed between the groups. In the first step, the predictors were estimated simultaneously for both groups using multi-group path analysis, and the construct validity of the model was tested; in the second step, the Wald test was used to determine whether the magnitudes of the associations of the predictors found to be significant in both groups differed statistically between the groups. Because PISA employs a complex, stratified, and clustered sampling design, the analyses followed the procedures recommended in the PISA Data Analysis Manual (OECD, 2009) and the PISA 2022 Technical Report (OECD, 2024). First, all parameter estimates were computed using the final student weight (W_FSTUWT), so that the estimates reflect the sampling design rather than the unweighted sample. Second, standard errors were estimated with the balanced repeated replication (BRR) method using Fay (1989) modification (Fay coefficient = 0.5) and the 80 replicate weights (W_FSTURWT1–W_FSTURWT80) provided in the database; this approach accounts for the design effects introduced by stratification and clustering, which conventional standard errors ignore (OECD, 2024). Third, to incorporate the measurement uncertainty of the achievement estimates, the model was estimated separately for each of the ten plausible values and the results were combined using Rubin (1987) rules, so that both the sampling variance and the between-imputation variance were reflected in the reported standard errors (Rutkowski et al., 2010). Furthermore, since the data set violated the assumption of multivariate normality, the Maximum Likelihood Robust (MLR) estimator, which allows for the estimation of parameters with the least error, was used (Muthén et al., 2017; Yuan and Bentler, 2000).

In the first step, all seventeen predictors were simultaneously included in the model for both groups, and the regression coefficients for each group were estimated independently (Model A). Since this structure results in a fully saturated model (*df* = 0), the perfect fit indices stem from a mathematical necessity; it is emphasized that evaluations at this stage should focus directly on the direction, magnitude, and statistical significance of the path coefficients (Byrne, 2013; Kline, 2023). To overcome the structural constraint arising from this model characteristic and to confirm that the obtained results do not stem from a chance fit, a restricted model was defined in which all regression paths were constrained to be equal (*df* = 17) across groups (Model B). Whether this restriction impaired model fit was tested using the Satorra-Bentler scaled chi-square difference test (Δχ2). In the analysis, the Satorra-Bentler scaled chi-square difference between the constrained Model B and the saturated Model A was statistically non-significant (pooled *D*_2_ = 0.63, *df* = 17 and 499.1, *p* = 0.867), confirming that constraining the regression paths to be equal across groups did not worsen model fit. The test was computed separately for each plausible value and the resulting statistics were pooled with the D_2_ procedure (Li et al., 1991); the ten individual comparisons ranged from *p* = 0.511 to *p* = 0.909. In terms of information criteria, the constrained Model B (*AIC* = 5,560.7, *BIC* = 5,679.6) was in fact preferred over the saturated Model A (*AIC* = 5,580.8, *BIC* = 5,795.9), as both criteria favored the more parsimonious specification. Taken together, the non-significant difference between Models A and B indicates that imposing cross-group equality on the coefficients does not reduce model fit.

In the second step, the Wald test of parameter constraints was applied to test whether the magnitudes of the associations found to be significant in both groups differed between the groups. The Wald test evaluates a single parameter constraint at a time by testing the null hypothesis of equal coefficients across groups (Bollen, 2014; Tabachnick et al., 2007). For example, suppose a Wald test is conducted to determine whether the association between a predictor and academic performance differs across groups. A statistically significant Wald chi-square (χ2) value indicates that the magnitude of that association differs between obese and non-obese adolescents. Because this procedure is applied to seventeen paths, the possibility of inflated Type I error was addressed in two ways. First, the joint test described above provides an overall assessment that is unaffected by the number of individual comparisons. Second, the *p* values from the seventeen Wald tests were adjusted with the Benjamini–Hochberg procedure, which controls the false discovery rate (Benjamini and Hochberg, 1995). Both unadjusted and adjusted values are reported in Table 2. Exact *p* values and 95% confidence intervals are reported for every coefficient in place of significance thresholds.

**Table 2:** Coefficients and between-group comparisons by obesity status.

	Non-obese		Obese		Between-group difference		
Predictor	*B* (SE) [95% CI]	*p*	*B* (SE) [95% CI]	*p*	Δ (SE) [95% CI]	χ^2^(1)	*p* (*p*_FDR_)
Microsystem							
Bodyima	−0.104 (0.031) [−0.164, −0.043]	0.001	−0.218 (0.038) [−0.293, −0.144]	< 0.001	−0.115 (0.049) [−0.211, −0.018]	5.405	0.020 (0.341)
Curioagr	0.250 (0.035) [0.182,0.319]	< 0.001	0.268 (0.049) [0.171,0.364]	< 0.001	0.017 (0.063) [−0.105,0.140]	0.078	0.780 (0.859)
Persevagr	0.160 (0.039) [0.083,0.236]	< 0.001	0.123 (0.054) [0.017,0.230]	0.023	−0.036 (0.062) [−0.158,0.085]	0.348	0.556 (0.859)
Emocoagr	0.156 (0.039) [0.079,0.233]	< 0.001	0.146 (0.041) [0.065,0.227]	< 0.001	−0.010 (0.057) [−0.122,0.102]	0.031	0.859 (0.859)
Coopagr	−0.095 (0.036) [−0.166, −0.024]	0.009	−0.126 (0.055) [−0.233, −0.019]	0.021	−0.031 (0.072) [−0.172,0.110]	0.187	0.666 (0.859)
Psychsym	0.032 (0.035) [−0.036,0.100]	0.352	0.092 (0.044) [0.005,0.179]	0.039	0.060 (0.056) [−0.050,0.169]	1.134	0.287 (0.859)
Empatagr	0.021 (0.031) [−0.039,0.082]	0.491	−0.049 (0.043) [−0.133,0.036]	0.259	−0.070 (0.052) [−0.171,0.032]	1.821	0.177 (0.859)
Asseragr	−0.001 (0.024) [−0.047,0.046]	0.980	0.020 (0.034) [−0.048,0.087]	0.564	0.020 (0.040) [−0.059,0.100]	0.257	0.613 (0.859)
Stresagr	−0.038 (0.041) [−0.118,0.042]	0.352	0.042 (0.040) [−0.035,0.120]	0.284	0.080 (0.057) [−0.032,0.193]	1.961	0.161 (0.859)
Lifesat	−0.009 (0.042) [−0.091,0.073]	0.836	0.013 (0.062) [−0.109,0.135]	0.838	0.021 (0.072) [−0.120,0.163]	0.088	0.767 (0.859)
Mesosystem							
Soccon	−0.216 (0.035) [−0.285, −0.146]	< 0.001	−0.166 (0.051) [−0.265, −0.067]	0.001	0.050 (0.057) [−0.062,0.161]	0.761	0.383 (0.859)
Soconpa	0.045 (0.027) [−0.008,0.097]	0.095	0.059 (0.044) [−0.028,0.146]	0.186	0.014 (0.053) [−0.090,0.118]	0.068	0.794 (0.859)
Relatst	0.021 (0.039) [−0.056,0.098]	0.589	0.055 (0.059) [−0.061,0.172]	0.351	0.034 (0.070) [−0.103,0.171]	0.242	0.623 (0.859)
Exosystem							
Schrisk	−0.099 (0.034) [−0.165, −0.034]	0.003	−0.115 (0.047) [−0.208, −0.022]	0.015	−0.016 (0.056) [−0.126,0.095]	0.077	0.782 (0.859)
Feelsafe	0.083 (0.032) [0.020,0.146]	0.009	0.054 (0.053) [−0.050,0.158]	0.305	−0.029 (0.059) [−0.145,0.087]	0.242	0.623 (0.859)
Belong	−0.038 (0.042) [−0.121,0.045]	0.369	0.037 (0.067) [−0.095,0.169]	0.584	0.075 (0.082) [−0.087,0.237]	0.822	0.365 (0.859)
Bullied	−0.038 (0.028) [−0.093,0.017]	0.180	−0.025 (0.046) [−0.115,0.066]	0.590	0.013 (0.056) [−0.097,0.123]	0.053	0.819 (0.859)

*N* = 709 obese and 1,418 non-obese adolescents. The outcome is academic performance expressed as the standardized mean of the reading, mathematics, and science plausible values. All estimates are weighted by the final student weights; standard errors are obtained through balanced repeated replication with Fay's modification (Fay coefficient = 0.5, 80 replicate weights), and the ten plausible values are combined through Rubin's rules. Δ denotes the difference between the obese and non-obese coefficients, tested through a Wald statistic with one degree of freedom. Values in parentheses in the final column are *p* values adjusted for seventeen comparisons through the Benjamini–Hochberg procedure.

Because matching redefines the analytic sample, the coefficients describe associations within a configuration in which obese and non-obese adolescents are equated on ESCS, country, and gender, rather than within the unrestricted student population of the three systems. The estimates proved insensitive to this arrangement: repeating the analysis without the sampling weights changed no coefficient by more than 0.022, and the body image difference moved from −0.115 to −0.119. Three further checks pointed in the same direction. Excluding the forty students whose recorded BMI fell outside a plausible range, below 12 or above 60, left the body image difference essentially unchanged (−0.103). The difference was also of comparable magnitude in the three systems, with no evidence of between-country heterogeneity (*Q*(2) = 2.47, *p* = 0.292, *I*^2^ = 18.9%), and the fixed-effect pooled estimate (−0.117, *SE* = 0.056) matched the estimate obtained from the combined sample. Finally, no predictor showed a variance inflation factor above 2.0 in either group.

## 4 Results

The associations between the seventeen predictors and adolescents' academic performance, and the extent to which these associations differed by obesity status, were examined through multi-group path analysis. The findings are presented at three levels. The first level reports the joint comparison of the seventeen coefficients across groups. The second reports the comparison of each coefficient with its counterpart in the other group. The third presents associations observed within a single group, for which no between-group difference was established.

At the model level, constraining all seventeen paths to be equal across groups did not worsen model fit (pooled *D*_2_ = 0.63, *df* = 17 and 499.1, *p* = 0.867). At the path level, the findings (see Table 2) reveal that some variables show comparable associations with academic performance regardless of adolescents' weight status. One of these, curiosity (Curioagr), reflecting the desire for intellectual exploration, showed the strongest positive association with academic performance in both groups (non-obese: *B* = 0.250, *p* < 0.001; obese: *B* = 0.268, *p* < 0.001). Similarly, internal emotion regulation skills (Emocoagr; non-obese: *B* = 0.156, *p* < 0.001; obese: *B* = 0.146, *p* < 0.001) and perseverance, representing focus on long-term goals (Persevagr; non-obese: *B* = 0.160, *p* < 0.001; obese: *B* = 0.123, *p* = 0.023) were positively associated with academic performance for both student profiles. In contrast, school safety risks reflecting physical threats on school property (Schrisk; non-obese: *B* = −0.099, *p* = 0.003; obese: *B* = −0.115, *p* = 0.015), peer-oriented general social connections (Soccon; non-obese: *B* = −0.216, *p* < 0.001; obese: *B* = −0.166, *p* = 0.001), and the tendency to cooperate (Coopagr; non-obese: *B* = −0.095, *p* = 0.009; obese: *B* = −0.126, *p* = 0.021) were negatively associated with academic performance, regardless of weight status. For these six paths, the Wald tests provided no evidence that the coefficients differ by obesity status (χ^2^ (1) = 0.03 to 0.76, *p* = 0.383 to 0.859). The cooperation estimate warrants a methodological note. Its bivariate correlation with achievement was close to zero in each group (*r* = −0.002 and −0.008), and cooperation correlates moderately with several other predictors in the model, most notably sense of belonging, empathy, and life satisfaction (*r* = 0.30 to 0.36); the negative coefficient emerges only once this shared variance is partialled out. It is therefore interpreted as a feature of the adjusted model rather than as a substantive association between cooperation and achievement.

A key finding revealed by the multi-group path analysis is that body image perception (Bodyima) is the only variable whose coefficient differed between groups. A more positive body image was associated with lower academic performance in both groups (non-obese: *B* = −0.104, *p* = 0.001; obese: *B* = −0.218, *p* < 0.001), and the association was more than twice as strong among obese adolescents [Δ = −0.115, 95% CI (−0.211, −0.018), χ^2^ (1) = 5.405, *p* = 0.020]. This difference did not remain significant once the seventeen comparisons were taken into account (*p*FDR = 0.341), and it is therefore treated as an exploratory result. The analyses also revealed group-specific patterns, whereby a predictor was associated with academic performance in one group but not in the other. For example, psychosomatic symptoms (Psychsym), the frequency of somatic complaints over the past 6 months, were not associated with academic performance in the non-obese group (*B* = 0.032, *p* = 0.352) but were positively associated with it among obese adolescents (*B* = 0.092, *p* = 0.039). Conversely, the subjective perception of safety in school spaces (Feelsafe) was positively associated with academic performance in non-obese adolescents (*B* = 0.083, *p* = 0.009) but showed no such association in the obese group (*B* = 0.054, *p* = 0.305). For neither variable did the direct between-group test indicate a difference (Psychsym: χ^2^ *(*1) = 1.134, *p* = 0.287; Feelsafe: χ^2^ (1) = 0.242, *p* = 0.623), and both confidence intervals for the difference included zero; these patterns are therefore reported as subgroup-specific associations rather than as differences between groups. On the other hand, the quality of student-teacher relationships (Relatst), sense of belonging to school (Belong), exposure to peer bullying (Bullied), social connection to parents (Soconpa), empathy (Empatagr), assertiveness (Asseragr), stress resistance (Stresagr), and life satisfaction (Lifesat) were not associated with academic performance in either group. The paths with the largest coefficients in each group, together with the body-image comparison, are displayed in Figure 2.

**Figure 2:**
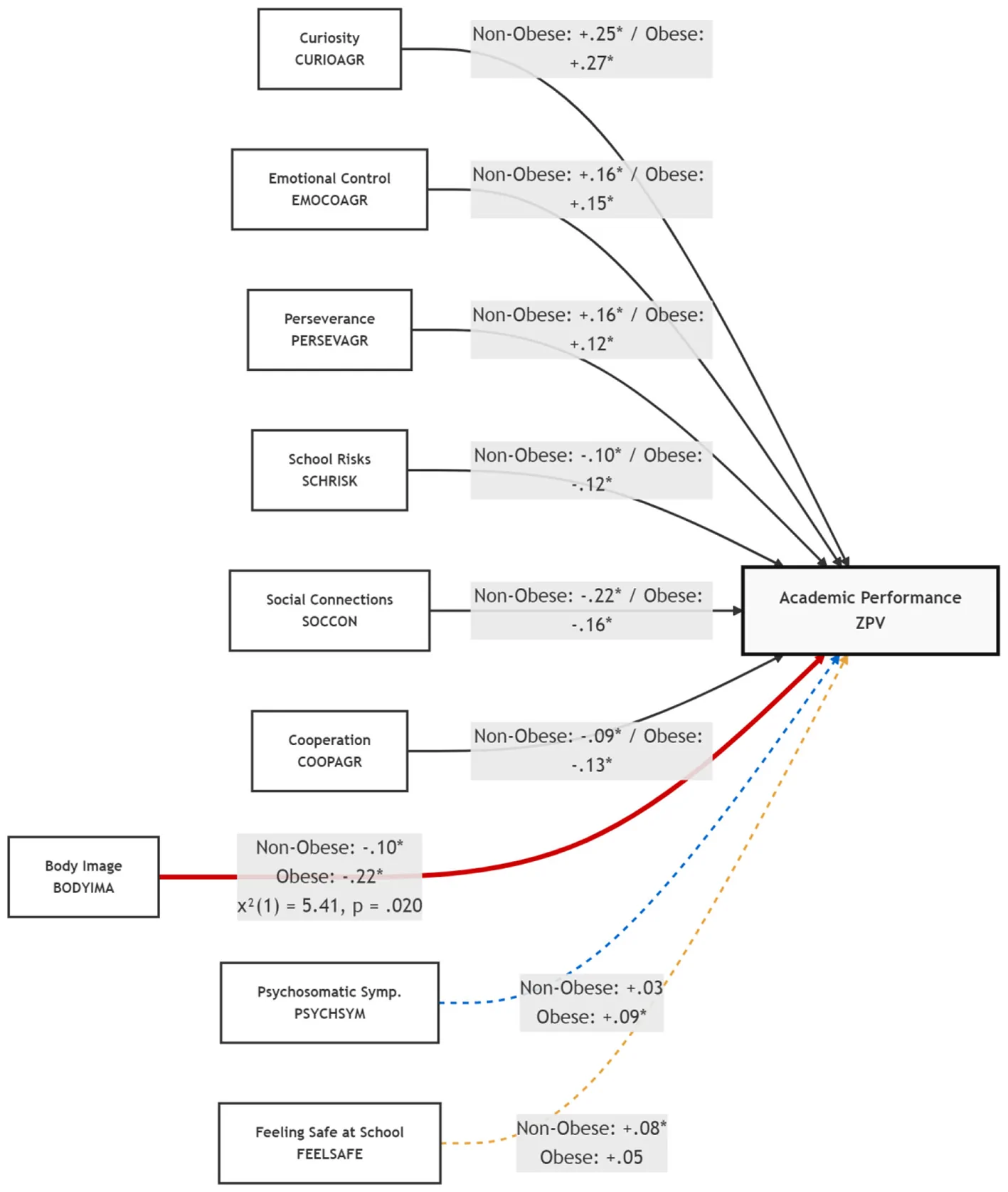
Multi-group path analysis structural diagram for predictors of academic performance. Coefficients are unstandardized and drawn from Table 2, where standard errors, confidence intervals, and exact *p* values are reported. Solid arrows denote paths that were significant in both groups and for which the Wald test indicated no between-group difference. The thick arrow marks the body image path, the only path whose coefficient differed between groups (χ^2^(1) = 5.41, *p* = 0.020); this difference did not remain significant after adjustment for the seventeen comparisons. Dashed arrows denote paths that reached significance in one group only, with no between-group difference established. Asterisks indicate coefficients significant at *p* < 0.05 within the group concerned.

## 5 Discussion

This study examined whether obesity moderates the associations between psychological and environmental resources and adolescents‘ academic performance within the framework of the Bioecological Model of Development (PPCT). The literature reviewed at the outset gives grounds to expect such moderation, particularly in the relational and institutional layers in which weight stigma is enacted. The present findings offer little support for that expectation. Sixteen of the seventeen associations were of comparable magnitude across groups, and no divergence was observed in any of the seven paths located in the mesosystem and the exosystem. The one exception falls within the microsystem, in how adolescents perceive their own bodies, and this difference did not persist once the seventeen comparisons were taken into account. What differs by weight status thus appears to be narrower than the bioecological framing would anticipate, and it is located in adolescents' evaluation of their own bodies rather than in the relational and institutional resources available to them.

### 5.1 Microsystem: self-perception, internal processes, and psychological dynamics

A striking finding at the microsystem level points in the opposite direction from what the literature would predict. Body image was negatively associated with academic performance in both groups: adolescents who reported being more satisfied with their bodies tended to perform less well, and this pattern was about twice as strong among obese adolescents. Most work in this area assumes the reverse, treating body-image dissatisfaction as a route to disengagement and lower achievement (Emon et al., 2024; Sikdar, 2021). The direction of the association is the more secure part of this result, since it holds in both groups and at both the bivariate and adjusted levels. The difference in magnitude between the groups is the less secure part: it was the only such difference among the seventeen comparisons, and it did not persist once those comparisons were taken into account. Crocker and Major (1989) stigma model offers one way to read this: when people feel devalued because of a stigmatized trait, one response is to work harder in an area where they can still succeed, and Major and O'Brien (2005) describe academic effort as one such area. For an obese adolescent, such devaluation is reinforced by how others respond to their weight (Včelarová et al., 2022), and academic performance may become the area where that pressure is directed. This reading is consistent with the pattern observed but goes beyond what the present measures can show, since neither weight stigma nor compensatory effort was assessed.

Psychosomatic symptoms showed a positive relationship with academic performance in both groups. This relationship was statistically significant only among obese adolescents, but the two groups did not differ from each other. Anxiety-related somatic complaints are usually treated as an obstacle to learning, so this pattern points to a different dimension of the psychosocial pressure associated with obesity. Consistent with the compensation strategy in (Crocker and Major 1989) stigma model, one interpretation of this pattern among obese adolescents is that academic effort may be sustained alongside, rather than despite, somatic strain. Such effort may come at a cost; experimental work has linked exposure to weight stigma to elevated cortisol reactivity (Schvey et al., 2014), and academic motivation maintained under chronic stress has been described as fragile and prone to burnout over time (Salmela-Aro and Upadyaya, 2014). Body image and psychosomatic symptoms thus point in the same direction, each relating to academic performance in a way the literature would not anticipate. Both relationships were stronger among obese adolescents, and for body image the difference between the groups was statistically detectable, though it did not survive adjustment for multiple comparisons.

On the other hand, it was found that social-emotional skills such as curiosity, emotional control, and perseverance are strong and common characteristics positively related to performance in both groups, regardless of weight status (Soto et al., 2024). This finding is theoretically significant in terms of the *Person* component of the PPCT model because while obesity reshapes many of the interactions an adolescent has with their environment, its bearing on internalized cognitive and emotional skills appears limited. In other words, resources such as curiosity and perseverance are relatively resilient to the pressure of stigmatization, and the fact that these skills relate to academic performance with equal strength in both groups indicates that they serve a structural function that is not easily eroded by obesity (Gutman and Schoon, 2013; Soto et al., 2024). The tendency toward collaboration, however, was negatively associated with academic performance in both groups; one interpretation is that time and cognitive energy allocated to individual academic tasks may shift toward group work. On this reading, resources directed toward collaboration may reduce what remains for individual academic work.

### 5.2 Mesosystem: immediate social environment, relational networks, and inter-system interactions

Contrary to the initial expectation, parental support was not significantly related to academic performance in either group. This suggests that, in this matched sample, the quality of the parent-child bond was not decisive for achievement, and earlier claims about its compensatory role should be treated with caution. Relatedly, it has been demonstrated longitudinally that weight-based comments and criticism from the family deepen psychological problems in adolescents (Anastasiadou et al., 2025); this finding suggests that the quality of family support may matter as much as its quantity.

Another finding that appears to contradict general expectations in the literature is that the social connections variable, which reflects adolescents' comfort in discussing their anxieties and concerns with those around them (family, peers), was significantly but negatively related to academic performance in both groups. Although at first glance one might expect that sharing problems would create psychological relief and thereby support academic performance, this finding can be explained by Wang and Eccles' (2012) observations regarding the nature of the search for social support during adolescence. (Wang and Eccles 2012) report that adolescents' constant search for comfort from their surroundings and the support they receive from peer networks can, at times, lead to unexpected negative consequences for behavioral and academic outcomes at school. In this context, as (Smetana et al. 2006) also emphasize, the constant focus on and repeated discussion of problems and negative emotions in friendships (co-rumination) may consume the mental energy and time that should go to individual academic tasks. Relational resources at school may therefore operate less uniformly than is often assumed; (Chen and Lu 2022) similarly found that classroom-level peer support improved peer acceptance for students in general while conferring no comparable benefit on adolescents with obesity.

The quality of student-teacher relationships, another type of relational network, was not significantly related to academic performance in either group. Although (Roorda et al. 2011) emphasized in their comprehensive meta-analysis that positive teacher attitudes are an important protective factor, particularly for disadvantaged and at-risk students, the results of this study do not align with that expectation. The primary reason for this may lie in the developmental and institutional context in which 15-year-old students find themselves. The specialization of courses in middle and high school, coupled with a reduction in the duration of emotional contact between teachers and students, may limit the contribution of individual teacher support. For obese adolescents in particular, the weight-based peer exclusion and stigmatization experienced in the school environment may be significant enough that the compassion or academic guidance a teacher might offer is not sufficient on its own to compensate for this social isolation (Branigan, 2017; Puhl et al., 2016). This may suggest that, rather than relying on teachers' individual efforts to engage obese adolescents in academic life, broader efforts to address the exclusionary peer culture that has permeated the school as a whole could be needed.

### 5.3 Exosystem: organizational environment and school climate

Perceived safety at school was positively related to academic performance among non-obese adolescents, while the coefficient in the obese group pointed in the same direction without reaching statistical significance; the two groups did not differ from each other. The weaker association in the obese group raises a question about the traditional boundaries of how schools understand safety, since perceived safety does not preclude exposure to subtle microaggressions, mocking attitudes toward the body, or exclusion from group activities. Steele and Aronson (1995) account of stereotype threat may be relevant here. When a student worries about being judged through a stereotype, part of their attention goes to that worry rather than to the task in front of them, and this concern persists regardless of how secure the building itself is. In a similar way, Baumeister and Leary (1995) belonging hypothesis suggests that the primary threat to an obese adolescent may be less an external physical risk than weight-based isolation directed at their identity. A school can be physically safe and still leave an adolescent exposed to judgment about their body, which may explain why the association was weaker in the obese group.

Contrary to expectations in the literature (Puhl and Heuer, 2009), neither exposure to peer bullying nor a sense of belonging at school was significantly related to academic performance in either group. The fact that a direct association between bullying and academic performance was not detected does not mean that this variable is unrelated; rather, its relationship with achievement may be an indirect one, involving internalized variables such as body image and psychosomatic symptoms. It has been shown that in adolescents exposed to weight-based bullying, the primary harm lies in the somatic and psychological burden created by the way this experience is internalized rather than in physical contact (Lessard, 2022). When evaluated within the framework of Baumeister and Leary (1995) need to belong theory, the weakening of an obese adolescent's bonds of belonging may similarly relate to academic performance through body image and rising stress symptoms. Indeed, as emphasized by Tekchandani et al. (2026), the association between obesity and academic outcomes is generally indirect, with anxiety, low self-esteem, and depression among the factors linked to it.

Other variables that were not significantly related to academic performance, namely empathy, assertiveness, stress resistance, and life satisfaction, should also be evaluated in this context. The fact that these variables were not significant does not mean that the traits in question are unrelated to academic performance. The common variance shared with the strong predictors included simultaneously in the model may have largely absorbed the independent explanatory contribution of these variables.

## 6 Limitations and future directions

Several limitations should be considered when interpreting these findings. First, the analytic sample was reduced considerably relative to the full PISA 2022 dataset. Much of this reduction reflects the fact that the Well-Being Questionnaire and objective BMI data were collected only in a limited number of systems, so the study necessarily draws on the subset of students for whom these measures were available. A further reduction followed from the 1:2 propensity-score matching, which was used to make the obese and non-obese groups comparable on gender, country, and socioeconomic status. This design choice involves a trade-off: it strengthens internal validity by reducing demographic confounding, but it also limits the extent to which the estimates can be generalized to the full adolescent population. The size of the matched sample also bears on the between-group comparisons. With the number of obese adolescents available, only a substantially larger difference than the one observed would have remained significant after adjustment for seventeen tests. The absence of significant differences after adjustment is therefore consistent with an absence of large differences, though smaller ones cannot be ruled out. Future work could revisit these questions using the broader sample to test whether the findings hold in the wider adolescent population. Second, as a consequence, the sample was drawn from only three educational systems (Hong Kong, Ireland, and New Zealand). These systems differ in language, culture, and schooling structure, but they do not represent the full range of national and cultural contexts in which weight-based stigma operates, and the findings should be generalized to other countries and education systems with caution. Within these systems, the students retained for analysis were on average somewhat higher-achieving than those excluded, and this should be borne in mind when considering how widely the estimates apply. Third, the psychosocial constructs were measured through self-reported PISA indices, and the design is cross-sectional. Two consequences follow. Some of the processes discussed here, such as the internalization of stigma or the role of chronic stress, were not measured directly, and were therefore used to interpret the findings rather than as tested claims. The direction of the associations also cannot be established: for any of the predictors examined, the relationship may run the other way, or both may be shaped by something not included in the model. For example, lower body satisfaction may accompany greater academic effort, as suggested here, but sustained academic effort may equally leave less room for the physical and social activity that shapes how adolescents see their bodies. Longitudinal designs with more direct measures would be needed to separate these possibilities.

## 7 Conclusion

This study examined, within the framework of the Bioecological Model of Human Development (PPCT), whether obesity moderates the associations of psychosocial competencies, relational contexts, and school climate with academic performance. Across seventeen paths and three ecological layers, the evidence for such moderation was limited. The associations were largely equivalent in the two groups, and the one difference that emerged did not persist once the number of comparisons was taken into account. The contribution of the study lies in locating where weight status does and does not make a difference across the ecological layers.

The study's main conclusion is that obesity did not operate as a general moderator of the associations examined here. Two findings nevertheless stand out, and both have to do with the adolescent's own experience. Adolescents who were more satisfied with their bodies tended to perform less well, and this pattern was stronger among obese adolescents, though not reliably so once the seventeen comparisons were taken into account. Psychosomatic symptoms showed a positive relationship with academic performance, reaching significance among obese adolescents alone. Read together, these patterns are consistent with an account in which academic achievement becomes the domain where some obese adolescents respond to weight-based pressure, at a cost that shows in their bodies. Such an account remains interpretive, since no mechanism was tested here, yet it points to a view of academic disadvantage in which the burden lies less in behavior or motivation than in how stigmatization is internalized. School climate offers a further observation: a sense of belonging was not associated with academic performance in either group, and perceived school safety reached significance only among non-obese adolescents. In neither case did the two groups differ. The protective role often attributed to institutional climate may therefore be narrower than the literature assumes.

In conclusion, this study suggests that the educational significance of obesity is better understood in terms of the psychological depth of stigmatization and the quality of institutional support than in terms of weight itself. Creating a fair academic environment for obese adolescents requires transforming the social and structural conditions that perpetuate weight-based disadvantage, rather than changing their weight status. The results point to the need for schools to reconsider their approach to obesity. This does not mean setting aside health-oriented approaches. Work grounded in the health belief model shows that perceived susceptibility and self-efficacy shape young people's intentions regarding weight management (Albasheer et al., 2024), adolescents' own accounts connect their beliefs about diet to their eating and activity patterns (Alqahtani, 2025), and structured consultations with health professionals have produced changes in dietary and activity behaviors among young people with overweight and obesity (Lutaud et al., 2023; Shouli et al., 2024). Alongside these, schools could introduce anti-bullying policies that target weight-based stigmatization, implicit bias training for teachers, and closer school-family partnerships (Langford et al., 2022; Puhl and Heuer, 2009; Wang and Sheikh-Khalil, 2014).

## Data Availability

The data that support the findings of this study are openly available in Mendeley Data at https://doi.org/10.17632/bss2pwtpc9.2.

## References

[B1] Albasheer O. Hakami N. Abdelwahab S. I. Alqassim A. Y. Alharbi A. Abdelmola A. O. . (2024). Utilisation of the health belief model to study the behavioural intentions relating to obesity management among university students: a cross-sectional study. BMJ Open 14:e079783. doi: 10.1136/bmjopen-2023-079783 38702084 PMC11086379

[B2] Alqahtani N. S. (2025). Saudi adolescents' perceptions of dietary contributions to obesity: a qualitative study. PLoS ONE 20:e0334407. doi: 10.1371/journal.pone.0334407 41237150 PMC12617880

[B3] Anastasiadou D. Tárrega S. Fornieles-Deu A. Sánchez-Carracedo D. (2025). Family-based weight stigma and psychological wellbeing of adolescents: a longitudinal analysis of recent vs. cumulative exposure. Front. Psychiatry 16:1623411. doi: 10.3389/fpsyt.2025.1623411 41113192 PMC12531152

[B4] Baumeister R. F. Leary M. R. (1995). The need to belong: desire for interpersonal attachments as a fundamental human motivation. Psychol. Bull. 117, 497–529. doi: 10.1037/0033-2909.117.3.497 7777651

[B5] Benjamini Y. Hochberg Y. (1995). Controlling the false discovery rate: a practical and powerful approach to multiple testing. J. R. Stat. Soc. Series B Stat. Methodol. 57, 289–300. doi: 10.1111/j.2517-6161.1995.tb02031.x

[B6] Benner A. D. Boyle A. E. Bakhtiari F. (2017). Understanding students' transition to high school: demographic variation and the role of supportive relationships. J. Youth Adolesc. 46, 2129–2142. doi: 10.1007/s10964-017-0716-2 PMC5693765 28776119

[B7] Bollen K. A. (2014). Structural equations with latent variables. New York, NY: John Wiley and Sons.

[B8] Branigan A. R. (2017). (How) does obesity harm academic performance? Stratification at the intersection of race, sex, and body size. Sociol. Educ. 90, 25–46. doi: 10.1177/0038040716680271 29593365 PMC5868982

[B9] Bronfenbrenner U. Morris P. A. (2006). “The bioecological model of human development,” in Handbook of child psychology: Vol. 1. Theoretical models of human development, eds. W. Damon and R. M. Lerner (793–828). Hoboken, NJ: Wiley.

[B10] Byrne B. M. (2013). Structural Equation Modeling with Mplus: Basic Concepts, Applications, and Programming. New York, NY: Routledge.

[B11] Chen D. R. Lu H. H. (2022). Social alienation of adolescents with obesity in classrooms: A multilevel approach. Journal of Adolescence, 94, 81–91. doi: 10.1002/jad.12001 35353406

[B12] Cornell D. Shukla K. Konold T. R. (2016). Authoritative school climate and student academic engagement, grades, and aspirations in middle and high schools. AERA Open 2:2332858416633184. doi: 10.1177/2332858416633184

[B13] Crocker J. Major B. (1989). Social stigma and self-esteem: the self-protective properties of stigma. Psychol. Rev. 96, 608–630. doi: 10.1037/0033-295X.96.4.608

[B14] Crosnoe R. Muller C. (2004). Body mass index, academic achievement, and school context: examining the educational experiences of adolescents at risk of obesity. J. Health Soc. Behav. 45, 393–407. doi: 10.1177/002214650404500403 15869112

[B15] Darling N. (2007). Ecological systems theory: the person in the center of the circles. Res. Hum. Dev. 4, 203–217. doi: 10.1080/15427600701663023

[B16] Durlak J. A. Weissberg R. P. Dymnicki A. B. Taylor R. D. Schellinger K. B. (2011). The impact of enhancing students' social and emotional learning: a meta-analysis of school-based universal interventions. Child Dev. 82, 405–432. doi: 10.1111/j.1467-8624.2010.01564.x 21291449

[B17] Emon H. H. Sarker S. Lima M. S. A. Tasnim F. A. Al Nabil A. Azam M. I. . (2024). Prevalence of overweight and obesity and their impact on academic performance and psychological wellbeing among university students in 2024 in Bangladesh. PLoS ONE 19:e0315321. doi: 10.1371/journal.pone.0315321 PMC11630606 39656712

[B18] Fay R. E. (1989). “Theory and application of replicate weighting for variance calculations,” in proceedings of the section on survey research methods (Alexandria, VA: American Statistical Association), 212–217.

[B19] Guimarães J. P. Fuentes-García J. P. González-Silva J. Martínez-Patiño M. J. (2023). Physical activity, body image, and its relationship with academic performance in adolescents. Healthcare 11:602. doi: 10.3390/healthcare11040602 36833137 PMC9957426

[B20] Gutman L. M. Schoon I. (2013). The impact of non-cognitive skills on outcomes for young people: A literature review. University of London, Institute of Education. Available online at: https://discovery.ucl.ac.uk/id/eprint/10125763/1/Gutman_Schoon_%202013%20Non-cognitive_skills_literature_review_.pdf (Accessed June 12, 2026).

[B21] Hennessy S. Bilker W. B. Berlin J. A. Strom B. L. (1999). Factors influencing the optimal control-to-case ratio in matched case-control studies. Am. J. Epidemiol. 149, 195–197. doi: 10.1093/oxfordjournals.aje.a009786 9921965

[B22] Hermassi S. Ketelhut S. Konukman F. Ayari M. A. Al-Marri S. Al Rawahi N. . (2024). Differences in physical activity, sedentary behavior, health-related physical performance indices and academic achievement: a comparative study of normal-weight and obese children in Qatar. J. Clin. Med. 13:1057. doi: 10.3390/jcm13041057 PMC10888728 38398370

[B23] Kline R. B. (2023). Principles and Practice of Structural Equation Modeling. New York, NY: Guilford Publications.

[B24] Langford R. Davies A. Howe L. Cabral C. (2022). Links between obesity, weight stigma and learning in adolescence: a qualitative study. BMC Public Health 22:109. doi: 10.1186/s12889-022-12538-w PMC8761050 35033056

[B25] Lessard L. M. (2022). Weight-based cybervictimization: implications for adolescent health. Pediatr. Obes. 17:e12888. doi: 10.1111/ijpo.12888 35076170

[B26] Li K. H. Meng X. L. Raghunathan T. E. Rubin D. B. (1991). Significance levels from repeated *p*-values with multiply-imputed data. *Statistica Sinica 1*, 65–92.

[B27] Lumeng J. C. Forrest P. Appugliese D. P. Kaciroti N. Corwyn R. F. Bradley R. H. (2010). Weight status as a predictor of being bullied in third through sixth grades. Pediatrics 125, e1301–e1307. doi: 10.1542/peds.2009-0774 20439599 PMC4174570

[B28] Lutaud R. Mitilian E. Forte J. Gentile G. Reynaud R. Truffet C. . (2023). Motivational interviewing for the management of child and adolescent obesity: a systematic literature review. BJGP Open 7:BJGPO.2022.0145. doi: 10.3399/BJGPO.2022.0145 37402547 PMC11176675

[B29] Ma L. Gao L. Chiu D. T. Ding Y. Wang Y. Wang W. (2020). Overweight and obesity impair academic performance in adolescence: a national cohort study of 10,279 adolescents in China. Obesity 28, 1301–1309. doi: 10.1002/oby.22801 32447840

[B30] Major B. O'Brien L. T. (2005). The social psychology of stigma. Annu. Rev. Psychol. 56, 393–421. doi: 10.1146/annurev.psych.56.091103.070137 15709941

[B31] Morales-Suárez-Varela M. Clemente-Bosch E. Peraita-Costa I. Llopis-González A. (2018). Socioeconomic status in adolescents: a study of its relationship with overweight and obesity and influence on social network configuration. Int. J. Environ. Res. Public Health 15:2014. doi: 10.3390/ijerph15092014

[B32] Muthén B. O. Muthén L. K. Asparouhov T. (2017). Regression and Mediation Analysis Using Mplus. Los Angeles, CA: Muthén and Muthén.

[B33] Myers A. Rosen J. C. (1999). Obesity stigmatization and coping: relation to mental health symptoms, body image, and self-esteem. Int. J. Obes. 23, 221–230. doi: 10.1038/sj.ijo.0800765 10193866

[B34] Nam S. Park J. (2021). The moderating effect of gender on the relationships between obesity, wellbeing, and stress perception in Korean adolescents. BMC Public Health 21:1836. doi: 10.1186/s12889-021-11894-3 PMC8518173 34649552

[B35] NCD Risk Factor Collaboration (NCD-RisC). (2024). Worldwide trends in underweight and obesity from 1990 to 2022: a pooled analysis of 3663 population-representative studies with 222 million children, adolescents, and adults. Lancet 403, 1027–1050. doi: 10.1016/S0140-6736(23)02750-2 PMC7615769 38432237

[B36] OECD (2009). PISA Data Analysis Manual: SPSS and SAS, 2nd Edn. Paris: OECDPublishing.

[B37] OECD (2024). PISA 2022 Technical Report. Paris: OECDPublishing.

[B38] Oraibi O. Somaili M. Daghriri K. Alameer M. Arishi M. Sahli A. . (2024). Investigating the interrelationships between obesity, academic achievement, physical activity, and social support among Jazan University students. Mater. Sociomed. 36, 59–64. doi: 10.5455/msm.2024.36.59-64 PMC10999139 38590594

[B39] Puhl R. M. Heuer C. A. (2009). The stigma of obesity: a review and update. Obesity 17, 941–964. doi: 10.1038/oby.2008.636 19165161

[B40] Puhl R. M. Himmelstein M. S. Pearl R. L. (2020). Weight stigma as a psychosocial contributor to obesity. Am. Psychol. 75, 274–289. doi: 10.1037/amp0000538 32053000

[B41] Puhl R. M. Neumark-Sztainer D. Bryn Austin S. Suh Y. Wakefield D. B. (2016). Policy actions to address weight-based bullying and eating disorders in schools: views of teachers and school administrators. J. Sch. Health 86, 507–515. doi: 10.1111/josh.12401 27246675

[B42] Roorda D. L. Koomen H. M. Spilt J. L. Oort F. J. (2011). The influence of affective teacher–student relationships on students' school engagement and achievement: a meta-analytic approach. Rev. Educ. Res. 81, 493–529. doi: 10.3102/0034654311421793

[B43] Rubin D. B. (1987). Multiple Imputation for Nonresponse in Surveys. New York, NY: Wiley.

[B44] Rutkowski L. Gonzalez E. Joncas M. Von Davier M. (2010). International large-scale assessment data: issues in secondary analysis and reporting. Educ. Res. 39, 142–151. doi: 10.3102/0013189X10363170

[B45] Salmela-Aro K. Upadyaya K. (2014). School burnout and engagement in the context of demands-resources model. Br. J. Educ. Psychol. 84, 137–151. doi: 10.1111/bjep.12018 24547758

[B46] Schvey N. A. Puhl R. M. Brownell K. D. (2014). The stress of stigma: exploring the effect of weight stigma on cortisol reactivity. Psychosom. Med. 76, 156–162. doi: 10.1097/PSY.0000000000000031 24434951

[B47] Shouli M. M. Shouli K. M. Aqtam I. Ayed A. (2024). Effect of nitro counseling on lifestyle of obese adolescence. SAGE Open Nurs. 10:23779608241228637. doi: 10.1177/23779608241228637 38322623 PMC10846033

[B48] Sikdar D. (2021). Effect of obesity on academic achievement and body image dissatisfaction among adolescents. Int. J. Multidiscip. Educ. Res. 10, 67–70.

[B49] Smetana J. G. Campione-Barr N. Metzger A. (2006). Adolescent development in interpersonal and societal contexts. Annu. Rev. Psychol. 57, 255–284. doi: 10.1146/annurev.psych.57.102904.190124 16318596

[B50] Soto C. J. Napolitano C. M. Sewell M. N. Yoon H. J. Roberts B. W. (2024). Going beyond traits: social, emotional, and behavioral skills matter for adolescents' success. Soc. Psychol. Personal. Sci. 15, 33–45. doi: 10.1177/19485506221127483

[B51] Steele C. M. Aronson J. (1995). Stereotype threat and the intellectual test performance of African Americans. J. Pers. Soc. Psychol. 69, 797–811. doi: 10.1037/0022-3514.69.5.797 7473032

[B52] Stuart E. A. (2010). Matching methods for causal inference: a review and a look forward. Stat. Sci. 25, 1–21. doi: 10.1214/09-STS313 20871802 PMC2943670

[B53] Tabachnick B. G. Fidell L. S. Ullman J. B. (2007). Using multivariate statistics. Boston, MA: Pearson.

[B54] Tekchandani N. Mukherjee A. Farook F. Varghese H. Aikoroje P. (2026). Understanding how childhood obesity influences educational outcomes: a literature review. Cureus 18:e101691. doi: 10.7759/cureus.101691 41700254 PMC12906851

[B55] Včelarová H. Cakirpaloglu P. Dostál D. Hladík J. (2022). Academic achievement, school absence and self-concept in Czech prepubescent children with overweight and obesity. Psychol. a Její Kontexty 13, 75–93. doi: 10.15452/PsyX.2022.13.0006

[B56] Wang M. T. Eccles J. S. (2012). Social support matters: longitudinal effects of social support on three dimensions of school engagement from middle to high school. Child Dev. 83, 877–895. doi: 10.1111/j.1467-8624.2012.01745.x 22506836

[B57] Wang M. T. Sheikh-Khalil S. (2014). Does parental involvement matter for student achievement and mental health in high school? Child Dev. 85, 610–625. doi: 10.1111/cdev.12153 24033259

[B58] World Health Organization (n.d.). BMI-for-age (5-19 years). Available online at: https://www.who.int/tools/growth-reference-data-for-5to19-years/indicators/bmi-for-age (Accessed June 12, 2026).

[B59] Yuan K. H. Bentler P. M. (2000). Three likelihood-based methods for mean and covariance structure analysis with non-normal missing data. Sociol. Methodol. 30, 165–200. doi: 10.1111/0081-1750.00078

